# Clinical efficacy of direct anterior approach vs. other surgical approaches for total hip arthroplasty: A systematic review and meta-analysis based on RCTs

**DOI:** 10.3389/fsurg.2022.1022937

**Published:** 2022-10-03

**Authors:** Zhongsheng Zhou, Yang Li, Yachen Peng, Jinlan Jiang, Jianlin Zuo

**Affiliations:** ^1^ Department of Orthopedics, China-Japan Union Hospital of Jilin University, Changchun, China; ^2^ Scientific Research Center, China-Japan Union Hospital of Jilin University, Changchun, China

**Keywords:** total hip arthroplasty, direct anterior approach, randomized controlled trial, meta-analysis, clinical efficacy analysis

## Abstract

**Background:**

Direct anterior approach (DAA) is an accurate technique for total hip arthroplasty (THA) through the muscle gap. Physicians who apply DAA believe that it accelerates patient recovery and results in lower rates of postoperative dislocation. However, the traditional surgical approach adherents believe that it is shorter and has fewer complications than DAA.

**Methods:**

We use the method of META analysis to organize and analyze the data of the randomized controlled studies (RCT) obtained after our screening. To compare the clinical efficacy of DAA approach and other surgical approaches for THA.

**Results:**

After rigorous screening, 15 RCT studies were included in our study, and data were extracted. The study included 1,450 patients from 15 RCTs, with a mean age of 63 years and a distribution of 52–67 years. Six weeks after the operation, the Harris hip score of the DAA approach improved by an average of 4.06 points (95% confidence interval (CI) 2.54 −5.59, *P* < 0.01, *I*^2^ = 45%, which can significantly improve the clinical efficacy of patients. However, the 0.61 points [95% confidence interval (CI) −1.13 −2.34, *P* > 0.01, *I*^2 ^= 0%] at 3 months and 1.49 points [95% confidence interval (CI) −1.65 −2.25, *P* > 0.01, *I*^2 ^= 0%] at 12 months postoperatively. In terms of dislocation rate, results show that the use of DAAs does not reduce Dislocation Rate with significant statistical heterogeneity among study groups (95% CI 0.18–2.94 *P* > 0.001, *I*^2 ^= 0%).

**Conclusion:**

The hip function of DAA was superior to posterolateral approach (PLA) and latera approach (LA) in the early days after hip replacement, especially within six weeks. However, at six months or more after surgery, the difference was not significant. The DAA did not show a lower rate of dislocation than other surgical approaches.

**Systematic Review Registration:**

https://www.crd.york.ac.uk/PROSPERO

## Introduction

DAA is simply the exposure of the hip joint through the Hunter interval between the tensor fascia latae (TFL) and Sartorius muscle. DAA only exposes the surgical field through the gap between the muscles, without essentially damaging or affecting the external rotation muscle groups of the lower extremities such as the piriformis muscle, the piriformis muscle, and the gluteus medias muscle (1). Only a portion of the rectus femoris reflex head is severed for easier visualization during the final hip exposure.

Proponents of DAA believe that due to the use of accurate intermuscular access, there will be minor trauma and more early recovery for the patient. Especially in terms of dislocation rates, they believe that DAA will be lower than traditional surgery (2–4). However, the unique complications associated with DAA have been published, for example, damage to the lateral femoral cutaneous nerve. And there are no data to suggest that the long-term improvement of DAA is superior to conventional surgical approach. Many studies have shown that the learning curve for true mastery of DAA techniques is steep (5). Most studies have found that the complication rate only decreases when a surgeon has experienced 100 hand speed procedures. This can take more than a year for many surgeons performing THA to reach such a high number of cases, which prevents them from genuinely mastering DAA (6, 7).

The aim of our study was to conduct a comprehensive search of the published literature and registered experiments. All randomized controlled trials comparing the DAA surgical approach with other surgical approaches were screened. The clinical efficacy of different surgical methods was verified by META analysis.

## Materials and methods

### Search strategies

Before we started our review, we had an agreement outlining our search strategy, inclusion and exclusion criteria, the results we wanted to analyze, and this systematic review was *a priori* registered with PROSPERO (CRD42020222077).

PubMed, EmBase, Cochrane library, Web of science, CNKI, China Biomedical Literature Database (CBM) from inception to 2022, with no language restriction. The keywords used in the search were as follows: “direct anterior approach” OR “anterior” OR “direct anterior” AND “lateral approach” OR “lateral” OR “posterior” OR” posterior approach” AND “total hip arthroplasty” OR “total hip replacement” OR “THA” OR “THR” OR “Arthroplasty”, Replacement, Hip” [Mesh]”.

### Inclusion criteria

(1) Population: Patients after unilateral primary THA.
(2) Interventions: THA using DAA approache, and the clinical effects were compared.
(3) Comparison: THA using other surgical approaches, and the clinical effects were compared.
(4) Outcomes: The randomised controlled experiment contains the data we need.
(5) Studies: Randomized controlled trial

### Exclusion criteria

(1) Non-randomized controlled studies.
(2) Duplicate documents in the database.
(3) Published META analysis.
(4) The experimental design is obviously unscientific.

### Outcome measures

Primary outcome measure requiring META analysis: Harris hip score (6 weeks, 3 months, and 1 year).

The secondary outcome measures were

(1) Operation time
(2) Number of days in hospital
(3) Postoperative dislocation rate
(4) Acetabular abduction, and anteversion.

### Data collection and analysis

#### Selection of the studies

Following the search strategy described above, two independent researchers searched the selected databases separately. All documents retrieved from the database are imported into professional document management software for the initial work of removing duplicate documents. Then, the titles and abstracts of all the articles were read by two researchers to delete the articles that did not meet the requirements. During this process, the research methods used in the literature were reviewed with emphasis. If the methodological description in the abstract is vague, the full text needs to be reviewed to ensure that the included literature is a randomized controlled trial using the scientific method. When encountering a lack of necessary information in the literature, contact the authors of the paper. The study was excluded if complete information could not be obtained. If two reviewers disagree on the article, a third reviewer is asked to join. If the disagreement persists, the article will be deleted.

### Data extraction and management

All data were extracted independently by two researchers using a data extraction table. The extracted data included the author of the article, publication date, journal name, type of study, sample size, and baseline data of patients; in addition, the grouping of studies and the final outcome indicators that needed to be included were included.

### Assessment of risk of bias in included studies

Risk of bias of all literature included in this study will be independently reviewed by two investigators. The Cochrane Manual of Systematic Reviews was used as the tool for this review. Areas for review included the following 6 items: (1) methods of randomizing patients (assessing selection bias), (2) methods of concealing assignment information (assessing selection bias), (3) blinding of patients and researchers (assessing program implementation bias), (4) blinding of final clinical outcome assessment (assessment of detection bias), (5) outcome data integrity assessment (assessment of data loss bias), (6) selective publication of results (assessment of publication bias).

### Statistical analysis

Data were analyzed and graphed using the Review Manager database (RevMan version 5.3) for this study. Means and standard deviations were used to evaluate continuous values, and mean differences were used to evaluate clinical outcomes for continuous values. Weighted mean differences were used to evaluate results between studies. Proportions or risk values were used to evaluate dichotomous values, and differences in risk were used to evaluate clinical outcomes for dichotomous values. Forest plots were used to show the final results. Studies with fixed effects models were evaluated using the Mantel-Haenszel (M-H) method. Q and I^2^ were used to assess the heterogeneity of studies, with *I*^2^ > 50% indicating heterogeneity between studies. Funnel plots were used to assess selective publication bias.

## Results

### Characteristics of the selected studies

**Figure 1:**
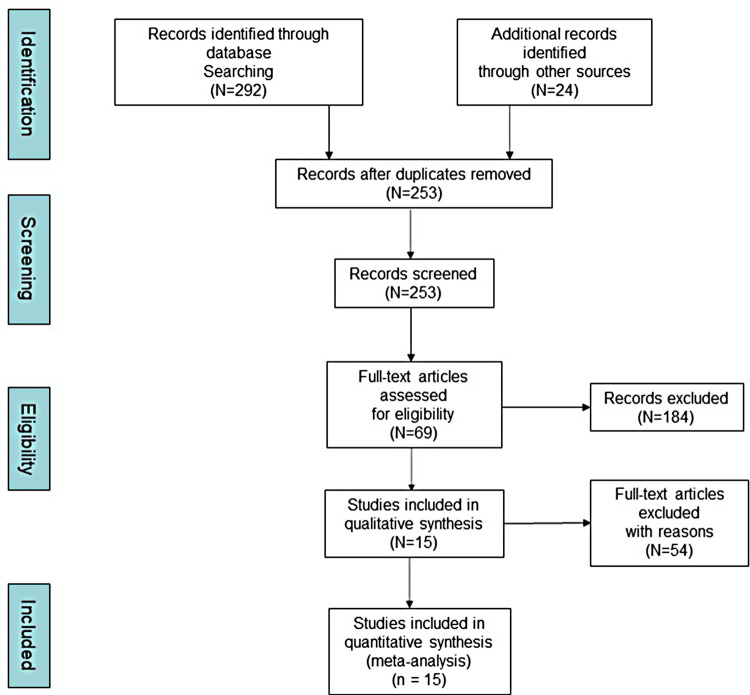
The processes of inclusion and exclusion (PRISMA).

316 articles were initially obtained; 63 duplicates were removed through screening. By reading the titles and abstracts of the included studies, 184 articles were removed, including case reports, animal experiments, and articles not relevant to the purpose of this study. After an initial screening, 69 studies were determined to be preliminary eligible. Two investigators then read the 69 articles in their entirety, and 48 articles were excluded because they could not be identified as RCTs. The other 3 articles were excluded because the required data could not be extracted. Three other articles were unanimously considered by the researchers to be inconsistent in the interventions and therefore could not be included in this study. Ultimately, 15 randomized controlled trials (8–22) were included in this study, details of which can be found in Table 1. The article inclusion and exclusion process is shown in Figure 1.

**Table 1:** Characteristics of the selected studies.

Study	Intervention	Number	Age	Female/Male	BMI	Type
Bon 2019	Anterior	50	67.26 ± 10^A^	29/21	26.46 ± 3.58	RCT
	Posterior	50	68.9 ± 7.93	23/27	26.69 ± 3.12	
Brismar 2018	Anterior	50	66 (58–74)^B^	32/18	27 (24–29)	RCT
	latera	50	67 (60–76)	33/17	27 (24–30)	
Mjaaland 2019	Anterior	84	67 ± 9	59/25	28 ± 4	RCT
	latera	80	66 ± 9	50/30	28 ± 4	
Parvizi 2016	Anterior	44	N/A	N/A	N/A	RCT
	latera	40	N/A	N/A	N/A	
Barrett 2013	Anterior	43	61.4 ± 9.2	14/29	30.7 ± 5.4	RCT
	Posterior	44	63.2 ± 7.7	25/19	29.1 ± 5.0	
Winther 2018	Anterior	20	56 (30–69)	15/5	25.8 ± 3.4	RCT
	Posterior	18	56 (44–67)	8/10	26.7 ± 3.7	
Rodriguez 2014	Anterior	60	N/A	N/A	N/A	RCT
	Posterior	60	N/A	N/A	N/A	
Müller 2012	Anterior	15	64.3 ± 7	9/6	26.9 ± 3.3	RCT
	latera	15	66.2 ± 8	10/5	27.0 ± 7.1	
Reichert 2018	Anterior	73	63.2 ± 8.2	32/41	28.3 ± 4.0	RCT
	latera	50	61.9 ± 7.8	26/24	28.7 ± 3.2	
Mjaaland 2015	Anterior	84	67.2 ± 8.6	59/25	27.7 ± 3.6	RCT
	latera	80	65.6 ± 8.6	50/30	27.6 ± 3.9	
Cheng 2017	Anterior	35	59 (54, 69)	20/15	27.7 (25.8,30.0)	RCT
	Posterior	38	62.5 (55,69)	20/18	28.3 (24.8,31.1)	
Martusiewicz 2020	Anterior	56	63 (41–83)	33/23	29.3 (19–35)	RCT
	Posterior	55	62 (49–79)	34/21	31.7 (21–43)	
Díaz 2016	Anterior	49	64.8 (10.1)	26/23	26.6 (3.9)	RCT
	latera	50	63.5 (12.5)	26/24	26.9 (3.1)	
Restrepo 2010	Anterior	50	62 (35-84)	N/A	25 (18.8-29.9)	RCT
	latera	50	59.9 (40,76)	N/A	25.1 (19.2,29.1)	
Bergin 2011	Anterior	29	68.8 9.1	17/12	26.3 ± 5.0	RCT
	Posterior	28	65.1 ± 11.3	14/14	27.8 ± 5.0	

N/A: not available A: Mean ± SD B: Median and inter-quartile range.

### Risk of bias in included studies

**Figure 2:**
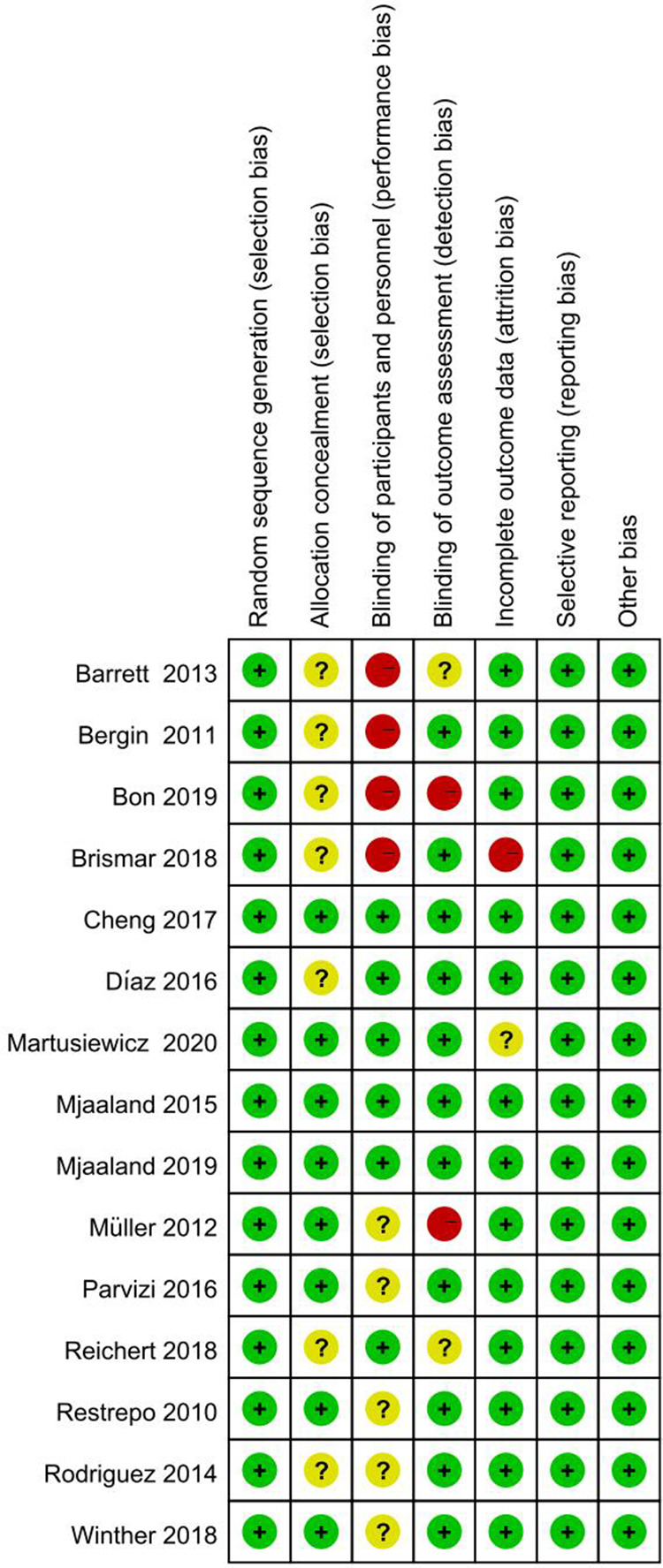
The quality assessment of each study.

**Figure 3:**
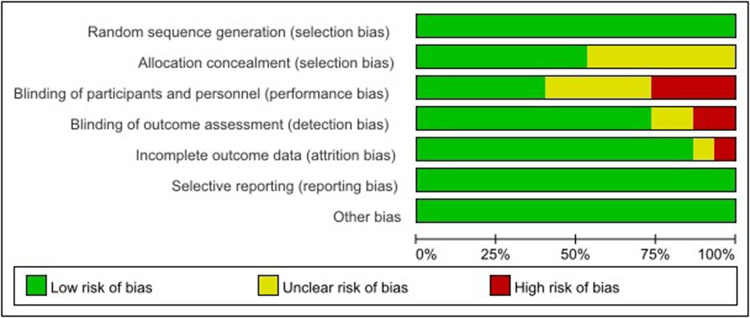
The quality assessment of each study.

Risk of bias for each study was independently assessed by two investigators. True double-blinding is difficult to achieve in this type of study, as surgeons inevitably know the procedure they are using. All 15 original papers included in the article were RCTs, and we evaluated the quality of the included papers, four of which excluded all bias. After a scientific evaluation by the investigators, 4 studies were selected as high-quality studies. Figures 2, 3 shows the results of the risk of bias assessment for all studies.

**Figure 4:**
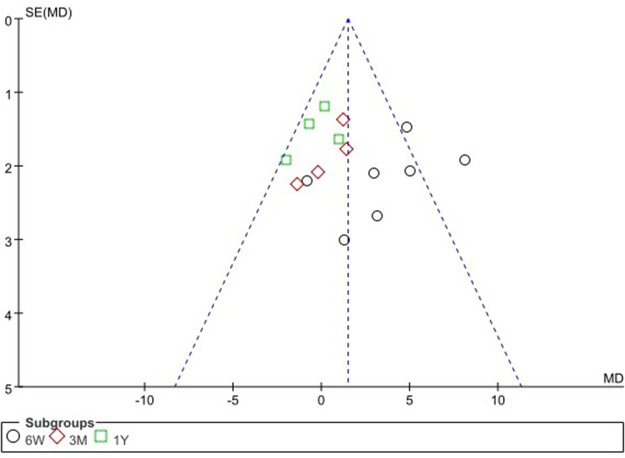
Direct anterior approach vs. other approaches: funnel plot of HHS.

We performed a funnel plot analysis of the primary outcome of this study, HHS score. Funnel plots can be used to assess publication bias in studies. The results of the analysis showed minimal asymmetry in pooled risk differences (RD), indicating a low risk of publication bias (Figure 4).

### Clinical efficacy analysis

HHS hip score will be the main analysis target of this study. Three different subgroups were divided according to time, and this was used to analyze the differences in HHS scores between DAA and other approaches over time. The secondary outcome measures include Operation time, Number of days in hospital, Postoperative dislocation rate, acetabular abduction and acetabular anteversion.

### HHS at 6 weeks、3 months、and 1 year

**Figure 5:**
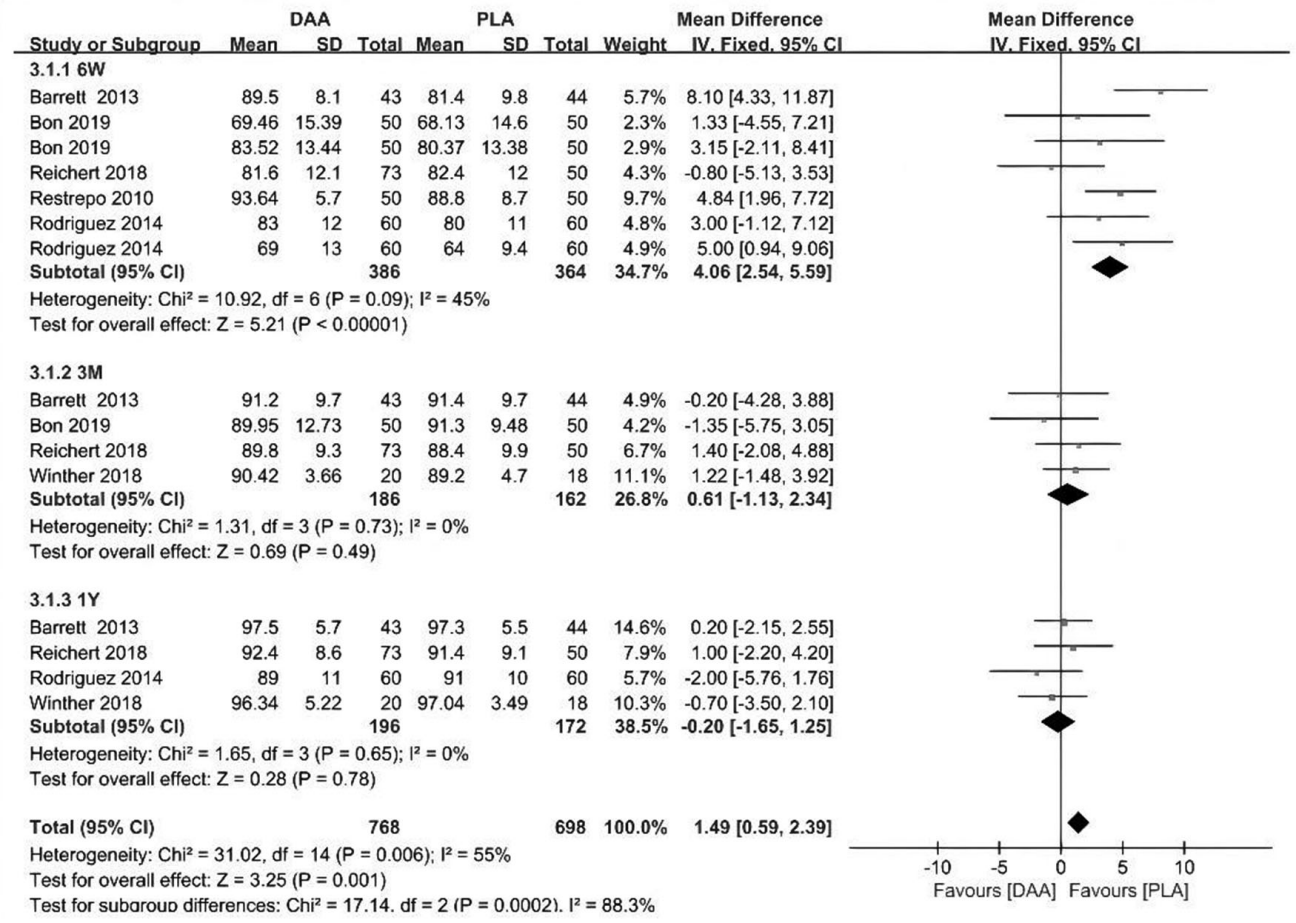
HHS outcome forest plot analyses.

Five studies gave HHS scores up to 6 weeks postoperatively, with two studies each including two sets of data, for seven sets of data included for comparison. The results showed that the HHS score was higher in the DAA group at 6 weeks after surgery, and there was no significant heterogeneity between the two groups in terms of statistics [MD 4.06, 95% confidence interval (CI) 2.54–5.59, *P* < 0.001, Heterogeneity *I*^2 ^= 45%]. However, there was no significant difference in HHS scores between the DAA and PLA groups at 3 months [MD 0.61, 95% confidence interval (CI) −1.13–2.34, *P* > 0.001, Heterogeneity *I*^2 ^= 0%] and 1 year [MD 4.06, 95% confidence interval (CI) −0.20–1.25, *P* > 0.001, Heterogeneity *I*^2 ^= 0%] postoperatively Figure 5.

### Surgery time

**Figure 6:**
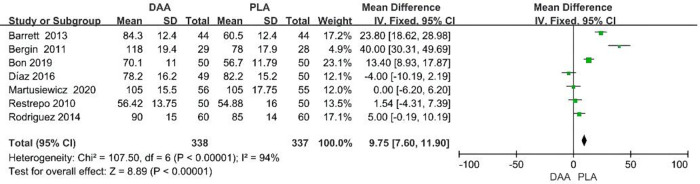
Surgery time outcome forest plot analyses.

In terms of operative time, data were extracted from 7 studies with a total of 675 patients. The results showed that when surgeons performed hip replacement using the DAA approach, the operating time was significantly increased. DAA increased operative time by a mean of 11 min (95% CI 1.8–20.31 *P* < 0.001, *I*^2 ^= 94%) Figure 6.

### Hospitalization time

**Figure 7:**
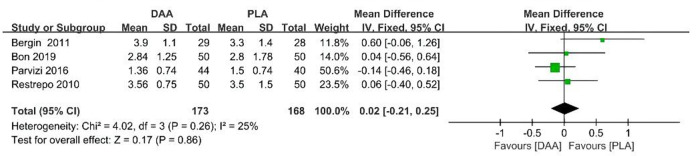
Hospitalization time outcome forest plot analyses.

During the data extraction process, hospitalization time data were extracted for a total of 351 patients from four studies. The results showed that the use of DAA did not reduce the length of hospitalization time (95% CI −0.21–0.25 *P* > 0.001, *I*^2 ^= 25%) Figure 7.

### Dislocation rate

**Figure 8:**
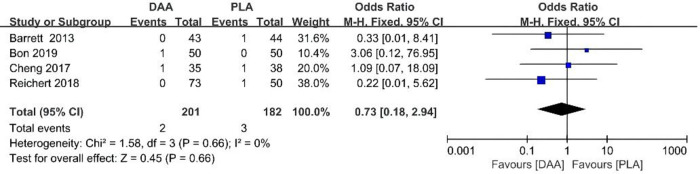
Dislocation rate outcome forest plot analyses.

During the data extraction process, dislocation rate data were extracted for a total of 383 patients from four studies. Results show that the use of DAAs does not reduce dislocation rate (95% CI 0.18–2.94 *P* > 0.001, *I*^2 ^= 0%) Figure 8.

### Acetabular abduction

**Figure 9:**
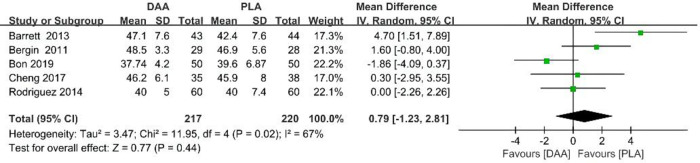
Acetabular abduction outcome forest plot analyses.

During the data extraction process, acetabular abduction data were extracted for a total of 417 patients from five studies. Results show that the use of DAAs does not reduce acetabular abduction (95% CI −1.23–2.81 *P* > 0.001, *I*^2 ^= 67%) Figure 9.

### Acetabular anteversion

**Figure 10:**
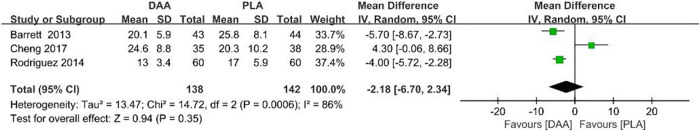
Acetabular anteversion outcome forest plot analyses.

During the data extraction process, acetabular anteversion data were extracted for a total of 280 patients from tfree studies. Results show that the use of DAAs does not reduce acetabular anteversion (95% CI −6.70–2.34 *P* > 0.001, *I*^2 ^= 86%) Figure 10.

## Discussion

The growing emphasis on minimally invasive arthroplasty and improved and expedited functional results make the direct anterior approach an attractive choice (23). Compared with the posterior approach, the lateral approach, and the anterolateral approach, it has certain advantages. For instance, studies have shown that patients undergoing DAA can perform well in recovery soon after surgery. Patients had a lower score on the visual analogue scale, a fashionable tool for the measurement of pain (24), on the first postoperative day, and more people performed well in climbing stairs and walking long distances in the 6th week. However, there was no significant difference at later time points (25). Research suggests that DAA, as a muscle-sparing THA surgical approach, may lead to longer operative times and more costs that are expensive (26). Additionally, studies have shown that DAA revision rates are higher in the early stages of the learning curve (27). Whether the clinical advantages of DAA (e.g., less soft tissue injury, less short-term postoperative pain, lower dislocation rates, and shorter hospital stay) are associated with higher costs compared to PA is open to question (26). In addition, in terms of the clinical efficacy of DAA, different researchers have different opinions. There is no clear evidence that hip replacement using DAA is superior to other surgical approaches in terms of long-term kinematic outcomes (28). Some researchers believe that DAA has a lower dislocation rate compared to PA and PLA (2–4). However, some researchers believe that compared with DAA, the overall complication rate of PA is significantly lower, and there is no difference in dislocation rate (29).

Although several studies have compared DAA with other surgical approaches, these studies include one network meta-analysis (30), two systematic reviews (28, 31), and seven meta-analyses (24, 25, 32–36). These studies have their own strengths and weaknesses, and our study can complement each other and greatly improve the quality of the included literature and the assessment of outcome measures. Fourteen RCTs were included in the network meta-analysis published by Putananon et al. (30), but the five most recently published RCTs were missing. At the same time, the study of Putananon et al. (30) did not include important indicators such as hospitalization time and operation time. In the studies by Kyriakopoulos et al. (31) and Meermans et al. (28), only a systematic review of the literature was conducted on different surgical approaches, and no quantitative meta-analysis was performed. Four of the studies included in the meta-analysis included non-randomized controlled studies, and these low-quality studies inevitably introduced bias (32, 34, 35). The remaining three articles were meta-analyses that included only RCT studies, of which Wang et al. (33), published in 2018, included nine RCT studies. Additional studies were included in our study due to the publication of new high-quality RCT studies after 2018. Therefore, our research is more comprehensive and more convincing. Published in 2019, Kucukdurmaz et al. (25) included 18 RCTs, more than ours. However, in Kucukdurmaz et al.'s study (25), only HHS scores were included at 6 weeks, and comparisons of results at longer follow-up were lacking. Furthermore, they did not include three recent high-quality RCTs. The Ramadanov et al. (36) study, published in 2021, included 10 RCTs. Their study, like Wang et al.'s (33) study, did not comprehensively incorporate all published RCTs. Furthermore, the Ramadanov et al. (36) study included THA resulting from fractures, which inevitably introduced bias. In conclusion, our study comprehensively included published RCT studies, all of which were hip replacements caused by hip non-fracture factors. Second, our study systematically compared important indicators including length of hospital stay. For the primary outcome measure, the HHS score, we analyzed data including one year to compare short-term and long-term clinical outcomes. Our results are more valuable for clinical guidance.

To make sensitive and repeatable measurements of hip surgery results, clinicians have designed many hip scoring systems to achieve this goal. Harris score is the most commonly used scoring system to evaluate THA procedures (37). It depends on the surgeon's judgment to assess the improvement of pain and short-term functional prognosis after THA (38, 39). Some studies have found that Harris hip scores have better average scores after using the direct anterior approach in the first six weeks after surgery. Subsequently, these scores did not differ from the posterior scores (8). This meta-analysis revealed that DAA had a significant improvement in HHS scores relative to PLA over six weeks. However, there were no significant differences in HHS scores at three months and one year postoperatively. This suggests that DAA can bring early recovery to patients, but there is no difference in long-term outcomes. In terms of operative time, however, as we would expect, DAA is longer than PLA, partly due to the need to use a traction bed, operator unskillfulness, and problems with the instrumentation itself, which may prolong the operative time. Still, in terms of time spent suturing the wound, DAA is significantly shorter than PLA, probably due to less tissue dissociation with DAA. An average of approximately 11 min is an acceptable increase in operative time. In terms of hospitalization time, the statistics show no difference between the two. This may have nothing to do with the DAA or PLA procedures. Instead, it depends on the bed turnover in the department and the personal habits of the surgeon (40).

Dislocation following total implantation of the hip joint is one of the common causes after TKA, which increases hospital costs by 300% of the expenditure of hip replacement alone. It is apparent that the causes of joint dislocation after hip replacement are relatively complex. Surgery-related factors may include the surgical approach, the angle of prosthesis placement, the size of the prosthesis. Patient-related factors included incorrect postoperative posture, history of Parkinson's disease, previous surgery, and accidents (41). There is an inherent perception that DAA may have a lower delocalization rate compared to other approaches. This study, however, does not support this intrinsic knowledge. The statistical results showed no statistical difference in the rate of dislocation and subsequent analyses of the acetabular abduction. Acetabular anteversion showed no statistical difference between the two, which coincidentally supports this conclusion. Some researchers consider DAA to be a surgical procedure performed through the natural muscle space (28, 42), which reduces the injury to the joint capsule and detachment of the surrounding muscles, resulting in a significant reduction in dislocation rates. However, some researchers have found that it may be the larger femoral component size that reduces this dislocation rate.

What's more, most surgeons with a direct anterior approach place the patient supine on a fracture table or regular table. The ability to perform dynamic testing (push-pull test) is lost during surgery on a specific operating table with a fixed limb. However, most surgeons of the posterior approach perform dynamic tests on the stability of the hip joint and the tension of the entire hip soft tissue during the operation (43). For example, intraoperative stability tests, especially the IR angle, were used to predict hip stability after THA (44).

This study confirms that DAA can bring early recovery to patients, but there is no difference in long-term outcomes, including dislocation rates. DAAs for hip replacement is becoming increasingly popular as patient demand for minimally invasive surgical techniques increases (45, 46). Modern surgical instruments combined with specially designed operating tables have made it easier for plastic surgeons to use the technique. However, the learning curve can be steep, requiring hundreds of conditions to become proficient. DAA is supported by many literatures and researchers for THA, but there is a lack of large randomized controlled studies to support this conclusion. Once a surgeon has mastered DAA, it is a viable alternative to performing successful surgical procedures.

## Data Availability

The original contributions presented in the study are included in the article/Supplementary Material, further inquiries can be directed to the corresponding author/s.

## References

[1] Nairn L Gyemi L Gouveia K Ekhtiari S Khanna V. The learning curve for the direct anterior total hip arthroplasty: a systematic review. Int Orthop. (2021) 45:1971–82. 10.1007/s00264-021-04986-7 33629172

[2] Sheth D Cafri G Inacio MC Paxton EW Namba RS. Anterior and anterolateral approaches for THA are associated with lower dislocation risk without higher revision risk. Clin Orthop Relat Res. (2015) 473:3401–8. 10.1007/s11999-015-4230-0 25762014 PMC4586236

[3] Fritz JK Waddell BS Kitziger KJ Peters PC Jr Gladnick BP. Is dislocation risk due to posterior pelvic tilt reduced with direct anterior approach total hip arthroplasty? J Arthroplasty. (2021) 36:3692–6. 10.1016/j.arth.2021.07.003 34330601

[4] Haynes JA Hopper RH Ho H McDonald JF Parks NL Hamilton WG. Direct anterior approach for primary total hip arthroplasty lowers the risk of dislocation compared to the posterior approach: a single institution experience. J Arthroplasty. (2022) 37:495–500. 10.1016/j.arth.2021.11.011 34774686

[5] Pirruccio K Evangelista PJ Haw J Goldberg T Sheth NP. Safely implementing the direct anterior total hip arthroplasty: a methodological approach to minimizing the learning curve. J Am Acad Orthop Surg. (2020) 28(22):930–6. 10.5435/JAAOS-D-19-00752 32015249

[6] Post ZD Orozco F Diaz-Ledezma C Hozack WJ Ong A. Direct anterior approach for total hip arthroplasty: indications, technique, and results. J Am Acad Orthop Surg. (2014) 22(9):595–603. 10.5435/JAAOS-22-09-595 25157041

[7] Siljander MP McQuivey KS Fahs AM Galasso LA Serdahely KJ Karadsheh MS. Current trends in patient-reported outcome measures in total joint arthroplasty: a study of 4 Major orthopaedic journals. J Arthroplasty. (2018) 33(11):3416–21. 10.1016/j.arth.2018.06.034 30057269

[8] Barrett WP Turner SE Leopold JP. Prospective randomized study of direct anterior vs postero-lateral approach for total hip arthroplasty. J Arthroplasty. (2013) 28(9):1634–8. 10.1016/j.arth.2013.01.034 23523485

[9] Bergin PF Doppelt JD Kephart CJ Benke MT Graeter JH Holmes AS Comparison of minimally invasive direct anterior versus posterior total hip arthroplasty based on inflammation and muscle damage markers. J Bone Joint Surg Am. (2011) 93(15):1392–8. 10.2106/JBJS.J.00557 21915544 PMC3143583

[10] Bon G Kacem EB Lepretre PM Weissland T Mertl P Dehl M Does the direct anterior approach allow earlier recovery of walking following total hip arthroplasty? A randomized prospective trial using accelerometry. Orthop Traumatol Surg Res. (2019) 105(3):445–52. 10.1016/j.otsr.2019.02.008 30853454

[11] Brismar BH Hallert O Tedhamre A Lindgren JU. Early gain in pain reduction and hip function, but more complications following the direct anterior minimally invasive approach for total hip arthroplasty: a randomized trial of 100 patients with 5 years of follow up. Acta Orthop. (2018) 89(5):484–9. 10.1080/17453674.2018.1504505 30350758 PMC6202757

[12] Cheng TE Wallis JA Taylor NF Holden CT Marks P Smith CL A prospective randomized clinical trial in total hip arthroplasty-comparing early results between the direct anterior approach and the posterior approach. J Arthroplasty. (2017) 32(3):883–90. 10.1016/j.arth.2016.08.027 27687805

[13] De Anta-Díaz B Serralta-Gomis J Lizaur-Utrilla A Benavidez E López-Prats FA. No differences between direct anterior and lateral approach for primary total hip arthroplasty related to muscle damage or functional outcome. Int Orthop. (2016) 40(10):2025–30. 10.1007/s00264-015-3108-9 26753844

[14] Martusiewicz A Delagrammaticas D Harold RE Bhatt S Beal MD Manning DW. Anterior versus posterior approach total hip arthroplasty: patient-reported and functional outcomes in the early postoperative period. Hip Int. (2020) 30(6):695–702. 10.1177/1120700019881413 31588801

[15] Mjaaland KE Kivle K Svenningsen S Nordsletten L. Do postoperative results differ in a randomized trial between a direct anterior and a direct lateral approach in THA? Clin Orthop Relat Res. (2019) 477(1):145–55. 10.1097/CORR.0000000000000439 30179928 PMC6345297

[16] Mjaaland KE Kivle K Svenningsen S Pripp AH Nordsletten L. Comparison of markers for muscle damage, inflammation, and pain using minimally invasive direct anterior versus direct lateral approach in total hip arthroplasty: a prospective, randomized, controlled trial. J Orthop Res. (2015) 33(9):1305–10. 10.1002/jor.22911 25877694

[17] Müller M Schwachmeyer V Tohtz S Taylor WR Duda GN Perka C The direct lateral approach: impact on gait patterns, foot progression angle and pain in comparison with a minimally invasive anterolateral approach. Arch Orthop Trauma Surg. (2012) 132(5):725–31. 10.1007/s00402-012-1467-x 22294091

[18] Reichert JC von Rottkay E Roth F Renz T Hausmann J Kranz J A prospective randomized comparison of the minimally invasive direct anterior and the transgluteal approach for primary total hip arthroplasty. BMC Musculoskelet Disord. (19 Jul. 2018) 19(1):241. 10.1186/s12891-018-2133-4 30025519 PMC6053824

[19] Parvizi J Restrepo C Maltenfort MG. Total hip arthroplasty performed through direct anterior approach provides superior early outcome: results of a randomized, prospective study. Orthop Clin North Am. (2016) 47(3):497–504. 10.1016/j.ocl.2016.03.003 27241374

[20] Restrepo C Parvizi J Pour AE Hozack WJ. Prospective randomized study of two surgical approaches for total hip arthroplasty. J Arthroplasty. (2010) 25(5):671–9.e1. 10.1016/j.arth.2010.02.002 20378307

[21] Rodriguez JA Deshmukh AJ Rathod PA Greiz ML Deshmane PP Hepinstall MS Does the direct anterior approach in THA offer faster rehabilitation and comparable safety to the posterior approach? Clin Orthop Relat Res. (2014) 472(2):455–63. 10.1007/s11999-013-3231-0 23963704 PMC3890195

[22] Winther SB Foss OA Husby OS Wik TS Klaksvik J Husby VS. Muscular strength and function after total hip arthroplasty performed with three different surgical approaches: one-year follow-up study. Hip Int. (2019) 29(4):405–11. 10.1177/1120700018810673 30421633

[23] Chen W Sun JN Zhang Y Zhang Y Chen XY Feng S. Direct anterior versus posterolateral approaches for clinical outcomes after total hip arthroplasty: a systematic review and meta-analysis. J Orthop Surg Res. (23 Jun. 2020) 15(1):231. 10.1186/s13018-020-01747-x 32576223 PMC7310458

[24] Jia F Guo B Xu F Hou Y Tang X Huang L. A comparison of clinical, radiographic and surgical outcomes of total hip arthroplasty between direct anterior and posterior approaches: a systematic review and meta-analysis. Hip Int. (2019) 29(6):584–96. 10.1177/1120700018820652 30595060

[25] Kucukdurmaz F Sukeik M Parvizi J. A meta-analysis comparing the direct anterior with other approaches in primary total hip arthroplasty. Surgeon. (2019) 17(5):291–9. 10.1016/j.surge.2018.09.001 30361126

[26] Berg AR Held MB Jiao B Swart E Lakra A Cooper HJ Is the direct anterior approach to THA cost-effective? A markov analysis. Clin Orthop Relat Res. (2022) 480:1518–32. 10.1097/corr.0000000000002165 35254344 PMC9278943

[27] Aggarwal VK Elbuluk A Dundon J Herrero C Hernandez C Vigdorchik JM Surgical approach significantly affects the complication rates associated with total hip arthroplasty. Bone Joint J. (2019) 101-b:646–51. 10.1302/0301-620x.101b6.Bjj-2018-1474.R1 31154834

[28] Meermans G Konan S Das R Volpin A Haddad FS. The direct anterior approach in total hip arthroplasty: a systematic review of the literature. Bone Joint J. (2017) 99-b:732–40. 10.1302/0301-620x.99b6.38053 28566391

[29] Maratt JD Gagnier JJ Butler PD Hallstrom BR Urquhart AG Roberts KC. No difference in dislocation seen in anterior vs posterior approach total hip arthroplasty. J Arthroplasty. (2016) 31:127–30. 10.1016/j.arth.2016.02.071 27067754

[30] Putananon C Tuchinda H Arirachakaran A Wongsak S Narinsorasak T Kongtharvonskul J. Comparison of direct anterior, lateral, posterior and posterior-2 approaches in total hip arthroplasty: network meta-analysis. Eur J Orthop Surg Traumatol. (2018) 28:255–67. 10.1007/s00590-017-2046-1 28956180

[31] Kyriakopoulos G Poultsides L Christofilopoulos P. Total hip arthroplasty through an anterior approach: the pros and cons. EFORT Open Rev. (2018) 3:574–83. 10.1302/2058-5241.3.180023 30595843 PMC6275850

[32] Yue C Kang P Pei F. Comparison of direct anterior and lateral approaches in total hip arthroplasty: a systematic review and meta-analysis (PRISMA). Medicine (Baltimore). (2015) 94:e2126. 10.1097/md.0000000000002126 26683920 PMC5058892

[33] Wang Z Hou JZ Wu CH Zhou YJ Gu XM Wang HH A systematic review and meta-analysis of direct anterior approach versus posterior approach in total hip arthroplasty. J Orthop Surg Res. (2018) 13:229. 10.1186/s13018-018-0929-4 30189881 PMC6127950

[34] Miller LE Gondusky JS Bhattacharyya S Kamath AF Boettner F Wright J. Does surgical approach affect outcomes in total hip arthroplasty through 90 days of follow-up? A systematic review with meta-analysis. J Arthroplasty. (2018) 33:1296–302. 10.1016/j.arth.2017.11.011 29195848

[35] Higgins BT Barlow DR Heagerty NE Lin TJ. Anterior vs. posterior approach for total hip arthroplasty, a systematic review and meta-analysis. J Arthroplasty. (2015) 30:419–34. 10.1016/j.arth.2014.10.020 25453632

[36] Ramadanov N Bueschges S Lazaru P Dimitrov D. A meta-analysis on RCTs of direct anterior and conventional approaches in total hip arthroplasty. Sci Rep. (2021) 11:20991. 10.1038/s41598-021-00405-4 34697357 PMC8546071

[37] Sali E Marmorat JL Gaudot F Nich C. Perioperative complications and causes of 30- and 90-day readmission after direct anterior approach primary total hip arthroplasty. J Orthop. (10 Aug. 2019) 17:69–72. 10.1016/j.jor.2019.08.006 31879477 PMC6919351

[38] Bergin PF Unger AS. Direct anterior total hip arthroplasty. JBJS Essent Surg Tech. (2011) 1(3):e15. 10.2106/JBJS.ST.K.00015 31321120 PMC6554081

[39] Heller GZ Manuguerra M Chow R. How to analyze the visual analogue scale: myths, truths and clinical relevance. Scand J Pain. (2016) 13:67–75. 10.1016/j.sjpain.2016.06.012 28850536

[40] Edwards PK Queen RM Butler RJ Bolognesi MP Lowry Barnes C. Are range of motion measurements needed when calculating the harris hip score? J Arthroplasty. (2016) 31(4):815–9. 10.1016/j.arth.2015.10.016 26639985

[41] Kalairajah Y Azurza K Hulme C Molloy S Drabu KJ. Health outcome measures in the evaluation of total hip arthroplasties–a comparison between the Harris hip score and the Oxford hip score. J Arthroplasty. (2005) 20(8):1037–41. 10.1016/j.arth.2005.04.017 16376260

[42] Kayani B Konan S Chandramohan R Haddad FS. The direct superior approach in total hip arthroplasty. Br J Hosp Med. (2019) 80(6):320–4. 10.12968/hmed.2019.80.6.320 31180766

[43] Rowan FE Benjamin B Pietrak JR Haddad FS. Prevention of dislocation after total hip arthroplasty. J Arthroplasty. (2018) 33(5):1316–24. 10.1016/j.arth.2018.01.047 29525344

[44] Lu Y Xiao H Xue F. Causes of and treatment options for dislocation following total hip arthroplasty. Exp Ther Med. (2019) 18:1715–22. 10.3892/etm.2019.7733 31410129 PMC6676097

[45] Meneghini RM. Investigation of the unstable total hip arthroplasty. J Arthroplasty. (2018) 33(5):1325–7. 10.1016/j.arth.2018.01.052 29523442

[46] Tanino H Sato T Nishida Y Mitsutake R Ito H. Hip stability after total hip arthroplasty predicted by intraoperative stability test and range of motion: a cross-sectional study. BMC Musculoskelet Disord. (2018) 19:373. 10.1186/s12891-018-2289-y 30322394 PMC6190554

